# Integrated MALDI-MS imaging and LC–MS techniques for visualizing spatiotemporal metabolomic dynamics in a rat stroke model

**DOI:** 10.1007/s11306-013-0588-8

**Published:** 2013-10-13

**Authors:** Miho Irie, Yoshinori Fujimura, Mayumi Yamato, Daisuke Miura, Hiroyuki Wariishi

**Affiliations:** 1 Graduate School of Bioresource and Bioenvironmental Sciences, Kyushu University, 6-10-1 Hakozaki, Higashi-ku, Fukuoka, 812-8581 Japan; 2 Innovation Center for Medical Redox Navigation, Kyushu University, 3-1-1 Maidashi, Higashi-ku, Fukuoka, 812-8582 Japan; 3 Bio-architecture Center, Kyushu University, 6-10-1 Hakozaki, Higashi-ku, Fukuoka, 812-8581 Japan; 4 Faculty of Arts and Science, Kyushu University, 6-10-1 Hakozaki, Higashi-ku, Fukuoka, 812-8581 Japan

**Keywords:** MSI, LC–MS, Pathological analysis, Metabolomic dynamics, Spatiotemporal behavior, Stroke

## Abstract

Spatiotemporal information about biomolecules is indispensable for precise pathological analysis, but it remains largely unclear. Here we show a novel analytical platform combing mass spectrometry imaging (MSI) with its complementary technique, liquid chromatography–mass spectrometry (LC–MS), to elucidate more comprehensive metabolic behaviors, with spatiotemporal information, in tissues. Analysis of a rat transient middle cerebral artery occlusion (MCAO) brain tissue after ischemia–reperfusion was performed to characterize the detailed metabolomic response to pathological alterations. To compare the spatially resolved metabolic state between ischemic and contralateral hemispheres of the MCAO brain, coronally sliced tissues were subjected to MSI. We also measured the metabolites extracted from three different cerebral regions, including whole cortex (CTX), hippocampus (HI) and corpus striatum (CPu), by LC–MS. In the ischemic hemisphere, significant metabolic changes at the CTX and CPu were observed after reperfusion, while not at the HI. A region-specific metabolic behavior was observed in amino acid and nucleotide metabolism, as well as in the TCA cycle. Correlation between MSI and LC–MS data was relatively high in the CTX and CPu. Combination of both MS platforms visualized the diverse spatiotemporal metabolic dynamics during pathological progress. Thus, our proposed strategy will contribute to the understanding of the complex pathogenesis of ischemia–reperfusion.

**Electronic supplementary material:**

The online version of this article (doi:10.1007/s11306-013-0588-8) contains supplementary material, which is available to authorized users.

## Introduction

Stroke is the third major cause of death in the major industrialized countries, and is a brain disease associated with cell death due to a decrease in oxygen and glucose resulting from a lack of blood flow (ischemia) (Green 2008). The symptoms of this disease are caused by both blood flow blockage-induced occlusion injury and oxidative stress-induced reperfusion injury (Memezawa et al. 1992b; Martin 2003). To date, several therapeutic agents, including edaravone, have been developed to lessen the ischemia-induced damage. However, the efficacy of such agents was limited for treating acute ischemic stroke patients, because of the limited time window (~3 h after ischemia) (Zhang et al. 2005). Therefore, the development of drugs with a longer or later time window or that function via other mechanisms is necessary.

Previous research addressing the mechanisms of disease, including stroke, have been targeted to particular genes, proteins and metabolites, and the analysis of only a part of the functions related to these biomolecules has been the focus of most investigations (Phillis et al. 1994, 1996). However, the data obtained from such studies is insufficient to understand the complex disease processes using conventional techniques alone, because various other, untargeted, biomolecules may participate in the pathological progress (Jung et al. 2011). Behaviors of biomolecules are drastically altered by the time elapsed from disease onset, and there are often local differences within the lesion site (Matsumoto et al. 1993). Therefore, to obtain a precise understanding of the biological complexity of disease progression, it is necessary to obtain more comprehensive and spatiotemporal information about the biomolecules in the target site.

Recently, “omics” technology, the analysis of comprehensive biomolecules, has received considerable attention. In particular, metabolomics, the analysis of comprehensive metabolites as a compound-level phenotype of genomic information, can elucidate the biological phenomena unresolved by transcriptomics and proteomics (Griffin and Shockcor 2004; Holmes et al. 2008; Nicholson et al. 1999). Generally, mass spectrometry (MS), coupled with pre-separation techniques such as liquid chromatography (LC)–MS or gas chromatography MS, has been used for the metabolomic studies due to their high coverage (more than several hundred metabolites) and ability to provide quantification (Dun and Ellis 2005; Werner et al. 2008; Major et al. 2006; Pohjanen et al. 2007). However, these methods have a drawback in the analysis of tissue samples because of the requirements for metabolite extraction, which causes the loss of information regarding the spatial localization of the metabolites. Visualization of the in situ interactions among a broad range of metabolites and their dynamic changes with information about their localization is indispensable for accurately understanding complex biological processes. In the pathological analysis of tissues with many functional compartments, such as the brain, it is necessary to not only determine the comprehensive metabolites involved in these processes, but also to visualize their spatial distribution within the tissue.

A remarkable new technology, MS imaging (MSI), enables the determination of the distribution of biomolecules present in tissue sections by direct ionization and detection (Caprioli et al. 1997; Miura et al. 2012). This technique can detect various biomolecules simultaneously without any labeling during a single analysis. Therefore, MSI is now being widely used for the in situ imaging of relatively abundant macromolecules, such as proteins, peptides and lipids, and is expected to be a potential tool for the pathological analysis of the brain, kidneys or tumors (Stoeckli et al. 2001; Amstalden et al. 2010; Vickerman 2011).

We recently succeeded in simultaneously visualizing more than 30 metabolites in normal mouse brain tissue by using matrix-assisted laser desorption/ionization-time of flight (MALDI-TOF)-MSI (Miura et al. 2010b). Moreover, the application of this technique and metabolic pathway analysis to a rat transient middle cerebral artery (MCA) occlusion (MCAO) model allowed for partially visualizing the spatiotemporal behavior of metabolites in the central metabolic pathway regulated by the ischemia–reperfusion insult (Miura et al. 2010b). This MSI technique is expected to be useful as an innovative multi-molecular imaging technique for high-precision pathological evaluation (Kinross et al. 2011). Therefore, an additional understanding of the dynamics of more comprehensive metabolites detected by LC–MS, which cannot be detected by MSI, may lead to further elucidation of the complex pathological mechanisms underlying various diseases.

In the present study, we performed LC–MS analysis of metabolites extracted from MCAO rat brain, and these data were co-analyzed with our previous and newly added MSI data (Miura et al. 2010b) to investigate the detailed metabolic dynamics during pathological progression. This approach could trace the diverse spatiotemporal metabolic behavior and visualize significant metabolic change in response to disease progression in the rat brain in a model of MCAO during infarct formation after ischemia–reperfusion.

## Materials and methods

### Materials

Indium tin oxide (ITO)-coated slide glass and 9-aminoacridine (9-AA) hydrochloride were obtained from Sigma-Aldrich (St Louis, MO, USA). The 9-AA was recrystallized prior to use. The organic solvents, internal standards and metabolite standards used in this study were purchased from Wako Pure Chemical Industries, Ltd (Osaka, Japan).

### Animal protocol

Male Wistar rats (6 weeks of age) were purchased from Kyudou (Tosu, Japan). The rats were housed in a temperature- and humidity-controlled room (23 °C, 78 % humidity), and fed a commercial diet (MF; Oriental Yeast, Tokyo, Japan) and water ad libitum. Reversible focal cerebral ischemia was induced with an intraluminal suture to produce a model of MCAO. In brief, anesthesia was induced with 2 % isoflurane (Dainippon Pharmaceutical, Osaka, Japan) in air, and was maintained with 1 % isoflurane using a facemask. A 1 cm midline incision was made on the anterior neck, and the right common carotid artery, external carotid artery and internal carotid artery were exposed. The common carotid artery and external carotid artery were ligated, and a suture was placed around the internal carotid artery for ligation. An embolus was made by inserting a 4–0 nylon surgical thread into the internal carotid artery through a small incision. The MCA was occluded by advancing the embolus into the internal carotid artery to block the origin of the MCA. After 1 h of MCAO, the MCA was reperfused by withdrawing the embolus. The rectal temperature was maintained by placing the rat on a heating pad under anesthesia. All the procedures and animal care were approved by the Animal Care and Use Committee, Kyushu University, and carried out in accordance with the Guidelines for Animal Experiments, Kyushu University. Extirpated tissue samples were immediately frozen and stored at −80 °C prior to use.

### MSI analysis of brain sections

**Fig. 1:**
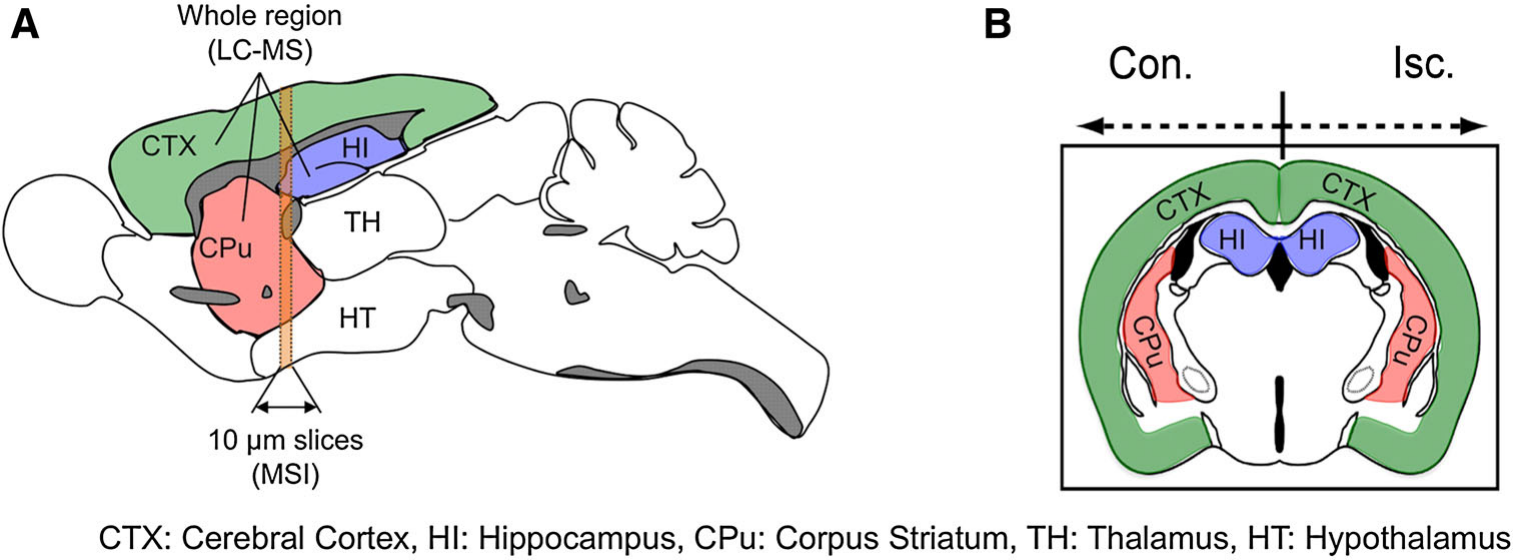
A schematic diagram of the sampling areas for the MSI and LC–MS analyses. Rat brain tissue samples were collected 0, 3 and 24 h after reperfusion following 1 h of MCAO. A schematic illustration represents the structures of the sagittally (**a**) and coronally (**b**) sectioned brains. In both the contralateral (Con.) and ischemic (Isc.) hemispheres, the whole cerebral cortex (CTX), hippocampus (HI) and corpus striatum (CPu) were enucleated from the brain for the LC–MS analysis. Coronal brain sections, including the CTX, CPu and HI, were subjected to the MSI analysis

In this study, we used three types of MS instruments. For MSI, single reflectron-type MALDI-TOF-MS (AXIMA Confidence, Shimadzu) was used. And For identification of metabolites by MS/MS analysis, quadrupole ion trap (QIT)-type (AXIMA QIT, Shimadzu) and TOF/TOF-type (AXIMA Performance, Shimadzu) instruments were used. As shown in Fig. 1a, 10 μm thick frozen coronal sections were used for the analysis, as described previously (Miura et al. 2010b). In the MSI experiments, the data were acquired in negative ionization mode with 50 μm spatial resolution (10 laser shots/data point), and the signals between *m*/*z* 50 and 1,000 were collected. 9-AA exhibits very few matrix-derived interferences in the low-mass range, achieved great improvement for the sensitivity of metabolite analysis (detection potential with sub-femtomole sensitivity for various endogenous metabolites) that is advantageous for the simultaneous detection of a variety of cellular metabolites (Miura et al. 2010a; Yukihira et al. 2010). The acquired MSI data were processed with the freely available software program, BioMap (http://www.maldi-msi.org). For normalization of the imaging data, we first constructed an averaged mass spectrum image ranging from *m*/*z* = 50 to 1,000 over the whole region of the tissue section. Second, averaged mass intensity maps of the overall mass range were created. Third, averaged mass intensity maps divided the intensity maps of each peak. This means that the intensity of each peak in each pixel was divided by the average of the total ion count of each pixel. This normalization process is critical for allowing a more quantitative comparison of the MSI data acquired from different tissue sections, because MALDI ionization has been known to cause both spot-to-spot and sample-to-sample variation in the signal intensities based on the heterogeneity of matrix crystals. The signal intensity of each imaging data in the figure was represented as the normalized intensity.

### Metabolomic analysis of brain extracts by LC–MS

The rat brain samples were prepared 0, 3 and 24 h after reperfusion following 1 h of MCAO. Metabolites were extracted from the whole cortex (CTX), hippocampus (HI) and corpus striatum (CPu) in the ischemic (Isc.) and contralateral (Con.) hemispheres. Each tissue sample was homogenized in 80 % MeOH, including 10 μM sulfanilamide and 2 μM 4-aminoantipyrine to evaluate the extraction efficiency, on ice (50 mg per tissue in 1 mL vials) using dounce tissue grinders. After centrifugation at 15,000×*g* for 30 min at 4 °C, the supernatant was collected, and an equal volume of a 2:1 H_2_O/CHCl_3_ solution was added and further mixed vigorously for 30 s. Each sample was then centrifuged at 15,000×*g* for 20 min at 4 °C. After centrifugation, the aqueous layers were collected. A centrifugal evaporator (CVE-2000, EYELA, Tokyo, Japan) was used for solvent removal and sample concentration. The resultant samples were stored at −80 °C until the analysis. Samples were dissolved in 30 μL of 20 % acetonitrile, including 10 μM 4-hydroxybenzophenone as an internal standard on ice, prior to the LC–MS analysis (LCMS-IT-TOF, Shimadzu Corporation, Kyoto, Japan). The instrument was fitted with a pentafluorophenylpropyl column (Discovery HS-F5 column 250 × 4.6 mm, Supelco, Bellefonte, PA), ovened at 40 °C. The mobile phase conditions were as follows: linear gradient analysis with mobile phase A (H_2_O with 0.1 % formic acid), and mobile phase B (acetonitrile). After a 4 min isocratic run at 100 % eluting solvent A, the ratio of eluting solvent B was linearly increased to 35 % from 4 to 8 min and to 50 % from 8 to 12 min. The use of 50 % eluting solvent B was maintained for 5 min. The column was then washed with 100 % of eluting solvent B for 5 min, and column equilibration was carried out with 100 % eluting solvent A for 7 min. A 3 μL aliquot of the sample solution filtered through a 0.2 μm PTFE filter (EMD Millipore Corporation, Billerica, MA) was injected onto the column with a flow rate of 0.2 mL/min.

For the MS, the instrument was operated using an electrospray ionization source in both positive and negative ionization modes. The ionization parameters were: capillary voltage, 4.5 and −3.5 kV; the nebulizer gas flow, 1.5 L/min; the CDL temperature, 250 °C and the heat block temperature, 250 °C. Peak picking, alignment and normalization of the mass spectral data obtained by LC–MS were performed using the Profiling Solution software program (Shimadzu Corporation, Kyoto, Japan). Identified metabolites were quantified using authentic standards, and then the data were expressed as the ratios (ischemic hemisphere/contralateral hemisphere).

### Multivariate statistical analysis

A principal component analysis (PCA) was performed using the SIMCA-P+ software program (Version 12, MKS-Umetrics, Umeå, Sweden), allowing the visualization of LC–MS multivariate information. All variables were centered on the average and were normalized by dividing by the standard deviation (unit variance).

Heat maps were displayed using the freely available software program, MultiExperiment Viewer (http://www.tm4.org). The metabolite concentration data of LC–MS and/or mass peak intensity data of MSI were converted to the ratio of the Isc. hemisphere to the Con. hemisphere. A heat map analysis was performed on the data to visualize the metabolic dynamics.

## Results and discussion

### Experimental flow

In this study, we analyzed the metabolic dynamics in the brain of MCAO model rats during infarct formation after ischemia–reperfusion by integrating different MS techniques (MSI and LC–MS) (Fig. S1). To reveal the differences in metabolic variations in each functional tissue region of the brain, a metabolomic analysis of the CTX, CPu and HI regions was performed (Fig. 1). Rat brain samples were collected at different time intervals after ischemia–reperfusion (0, 3 and 24 h, *n* = 5) to investigate the time-dependent metabolic variations after reperfusion following 1 h of MCAO. To compare the metabolic state in the Isc. and Con. hemispheres of the MCAO brain (Fig. 1b), metabolites extracted from three different whole tissue regions (CTX, HI and CPu) were also measured by LC–MS. The CTX, CPu and HI tissue samples were coronally sectioned at 10-μm thickness, and metabolites on these partial tissue regions were directly measured by MSI to visualize their spatial distribution.

### Global investigation of region-specific metabolic dynamics during infarct formation

**Fig. 2:**
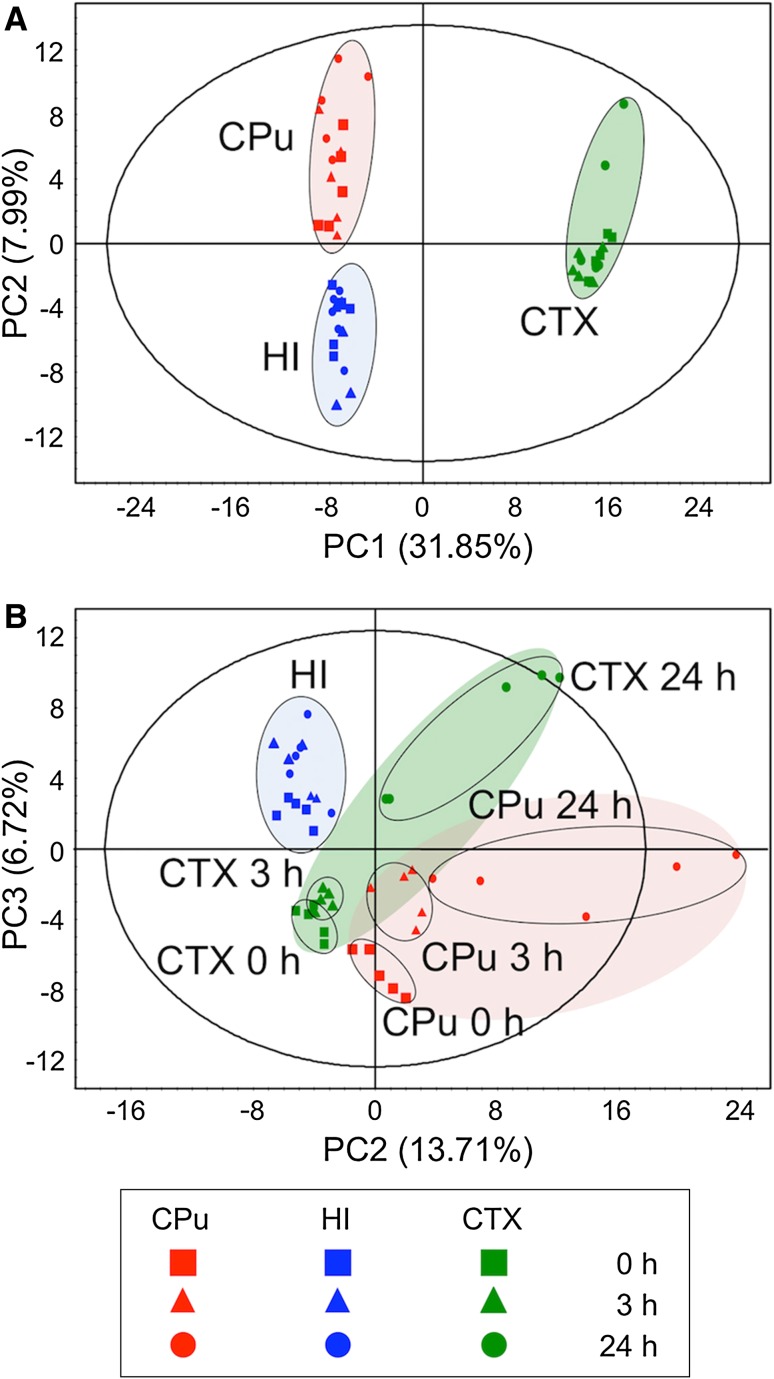
The multivariate analysis of cerebral metabolomic changes induced by reperfusion following 1 h MCAO. The CPu (*red*), HI (*blue*) and CTX (*green*) extracts from the contralateral (**a**) and ischemic hemispheres (**b**) at 0 h (*square*), 3 h (*triangle*) and 24 h (*circle*) after reperfusion were measured by LC–MS, and the resulting MS spectral data were subjected to an exploratory data analysis (PCA). The separate score plot is shown for each hemisphere’s data sets

In the present study, we performed a holistic evaluation of the metabolic profile in three regions (CTX, CPu and HI) and in each hemisphere (Isc. or Con.) at different times after reperfusion (0, 3 and 24 h) by a multivariate statistical analysis using LC–MS data sets (Fig. 2). We used an exploratory data analysis, PCA, which provides a summary or overview of all observations or samples in the data. In addition, groupings trends and outliers can be found easily. In the Con. hemisphere (Fig. 2a), the cluster formation of each region was observed, suggesting that the metabolic states were clearly different among the three representative compartments (CTX, CPu and HI). Such region-specific clusters were also formed in the Isc. hemisphere, and further time-dependent separation was observed in both the CTX and CPu regions, unlike the HI (Fig. 2b). These results indicate that the metabolic states of the CPu and CTX in the Isc. hemisphere were different from those in the Con. hemisphere, and that there were further time-dependent changes in response to ischemia–reperfusion injury. It is known that the MCA blood supply is important in both the CPu and CTX regions, but not in the HI region (Martin 2003; Kitagawa et al. 1998). This fact may provide insight into the region-specific metabolic alterations after ischemia–reperfusion.

**Fig. 3:**
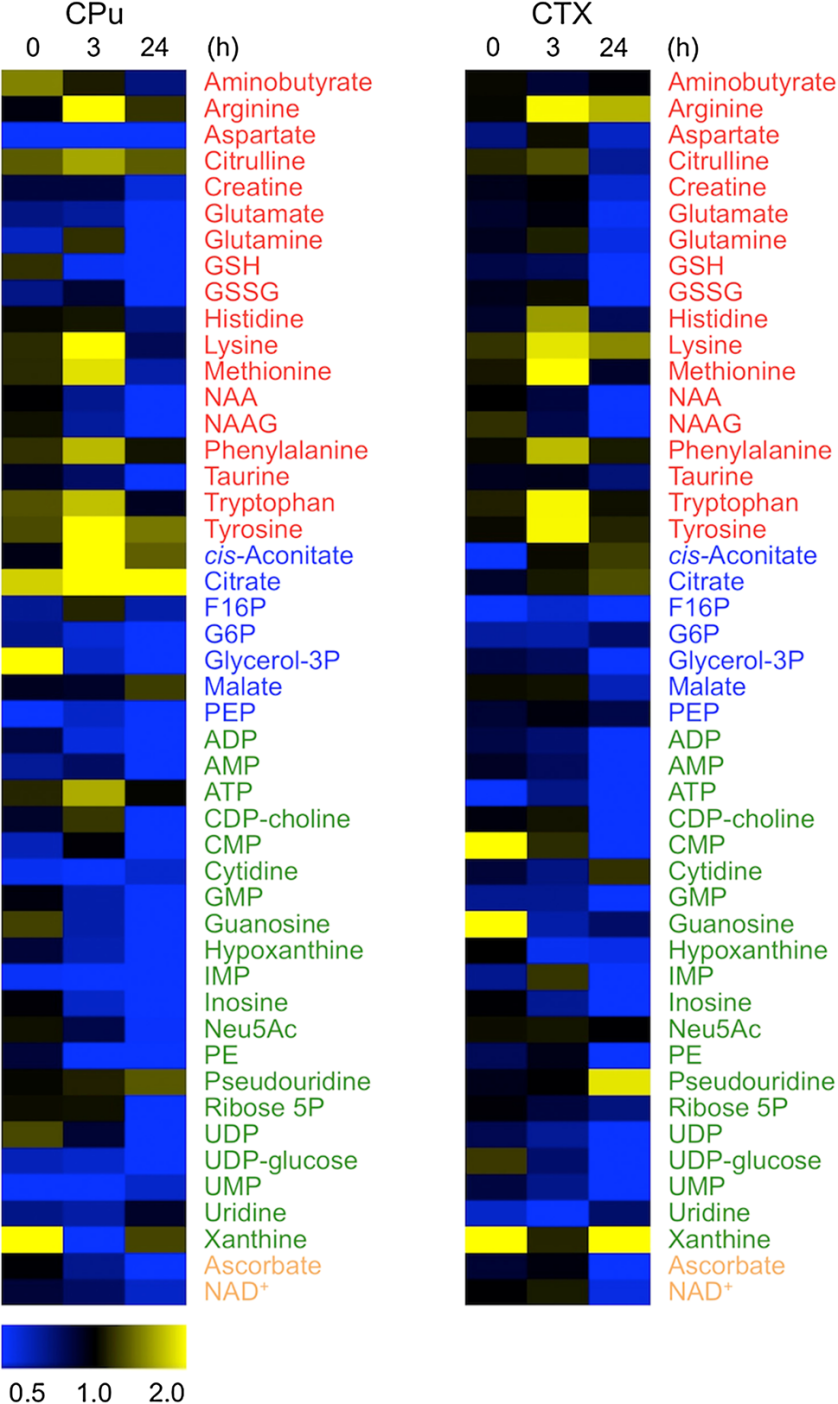
Heat map visualization of the common metabolites identified in two brain components during ischemia–reperfusion. Data were obtained by the average intensity ratio (ischemic hemisphere/contralateral hemisphere) from the LC–MS analysis. The data of 47 common metabolites detected in the CTX and CPu were visualized. The normalized mean values were displayed using the MultiExperiment Viewer (http://www.tm4.org). The colored letters for the heat map are as follows: amino acids (*red*), central metabolism intermediates (*blue*), nucleic acids (*green*), other metabolites (*orange*)

Next, we investigated the variations in each metabolite level in the CTX and CPu regions showing a significant metabolic change in response to disease progression. With regard to 47 common metabolites detected in both the CTX and CPu regions, changes of their ratio (Isc./Con.) at each time point were shown as a heat map (Fig. 3). The concentrations of almost all of the nucleic acids, except for ATP, UMP and CDP-choline in the CPu and ATP and IMP in the CTX, were reduced in a time-dependent manner. The peak accumulation of amino acids (e.g., lysine, phenylalanine, arginine and histidine) was observed at 3 h after reperfusion. The amounts of tricarboxylate cycle (TCA) cycle intermediates, such as citrate, in the Isc. hemisphere were sustained at higher levels compared to the Con. hemisphere. Although many of the detected metabolites were expressed at the same level in both hemispheres just after reperfusion, the difference in concentrations was clearly observed by 3 h after reperfusion. Especially at the time point 24 h after reperfusion, the majority of the metabolites in the CPu and CTX, except for pseudouridine and xanthine (malate in the CPu), were significantly reduced in the Isc. hemisphere compared with the Con. hemisphere.

A previous study reported that the production of reactive oxygen species (ROS) was especially increased at 1 or 2 h after reperfusion (Sims and Muyderman 2010; Lipton 1999). The generation of ROS causes an inactivation of metabolic enzymes and membrane lipid peroxidation (Martin 2003; Bromont et al. 1989). Therefore, reperfusion after 1 h MCAO may induce a failure of energy production and result in a low energy state in the cells present in the occluded MCA area (Sims and Muyderman 2010; Lipton 1999). Moreover, both the synthesis and degradation of proteins are dependent on the energy status (Bylund-Fellenius et al. 1984), and the enzymatic activity of protein synthesis was markedly inhibited after reperfusion (Abe et al. 1988). Hence, the above-mentioned alterations of the amino acid level may be due to an accumulation of amino acids as a result of the catabolism of proteins resulting from the inhibition of protein synthesis. Furthermore, it was already known that the activity of glucose-6-phosphate dehydrogenase in the pentose phosphate pathway, which generates nicotinamide adenine dinucleotide phosphate (NADPH) and ribose-5-phosphate for nucleotide synthesis, is reduced after reperfusion of the MCA (Yousuf et al. 2009; Sarkar and Das 2006). Such an alteration of enzymatic activity may cause a reduction in the overall nucleic acid level (Fig. 3). Taken together, these results raised the possibility that changes in the blood flow during MCA occlusion and reperfusion were associated with the region-specific metabolic behavior involved in the reduction of both energy production and related enzymatic activity.

Although many metabolite levels after reperfusion showed similar alterations in the CTX and CPu regions, the variation pattern of particular metabolites, such as malate, was clearly different (Fig. 3). A high concentration of citrate was sustained at the CPu in the Isc. hemisphere during reperfusion (0, 3 and 24 h), but the citrate level in the CTX showed a time-dependent accumulation. The metabolic response in the CTX was partially delayed compared with that in the CPu. A previous study reported that a decrease in the blood flow occurred over the whole region of the CPu following occlusion of the MCA, and that the medial area of the anterior CPu had relatively mild ischemia (blood flow rate: 50 %) (Memezawa et al. 1992a). On the other hand, the CTX is finely separated into several functional regions (Fig. S2) and its blood flow is supplied via multiple arteries, including the MCA. Therefore, the occlusion of the MCA causes a heterogeneous biological regulation in different functional regions of the CTX. The blood flow rate in the cingulate cortex was not significantly reduced by occlusion of the MCA, whereas the rates in the neocortex and piriform cortex were markedly decreased (Memezawa et al. 1992a). Moreover, the parietal cortex and somatosensory cortex, one of the neocortex-constructed regions, exhibited serious ischemia (blood flow rate: 15 %), while both other neocortex areas (e.g. the parietal cortex, motor area) and piriform cortex exhibited modest ischemia (blood flow rate: 20 %) (Memezawa et al. 1992a). These circumstances may thus help to explain the differences between the CPu and CTX regions in terms of the time-dependent metabolic dynamics during reperfusion.

### Comparison of the metabolic variations between the whole tissue region and its micro-regions in the MCAO rat brain after ischemia–reperfusion

Generally, an LC–MS analysis is able to detect and determine many kinds of metabolites, but the resulting data are averaged information from the whole tissue because of the extraction step. Tissue samples consisting of several functional compartments, such as brain, may have different metabolic regulation within smaller compartments contained within the single target region. Hence, the LC–MS technique may not correctly trace characteristic metabolic states during a pathological progress. To elucidate accurate and region-specific metabolic changes induced by ischemia–reperfusion, it is necessary to visualize the spatiotemporal metabolic behavior at the micro-regional scale.

To investigate spatially-resolved metabolic dynamics in micro-regions of the CPu and CTX, we used brain tissue sections (Fig. 1b) for MALDI-MSI, which enabled us to simultaneously detect multiple biomolecules with two-dimensional local information (Miura et al. 2010b). Although a part of the MSI data was already reported (Miura et al. 2010b), we compared the intensities of metabolite peaks between MSI and LC–MS. In the LC–MS analysis, the metabolites were extracted from whole CTX or CPu region in both the Isc. and Con. hemispheres, and the metabolite concentrations were averaged for each whole region (Fig. 1a). As shown in Fig. 1a, we prepared 10 μm brain slices, including the central portions of the ischemic lesions. The signal intensity of each metabolite within each region of interest (CTX (green) and CPu (red) as shown in Fig. 1b) was averaged, and then the data were used for the assessment of region-specific changes in the metabolite levels.

**Fig. 4:**
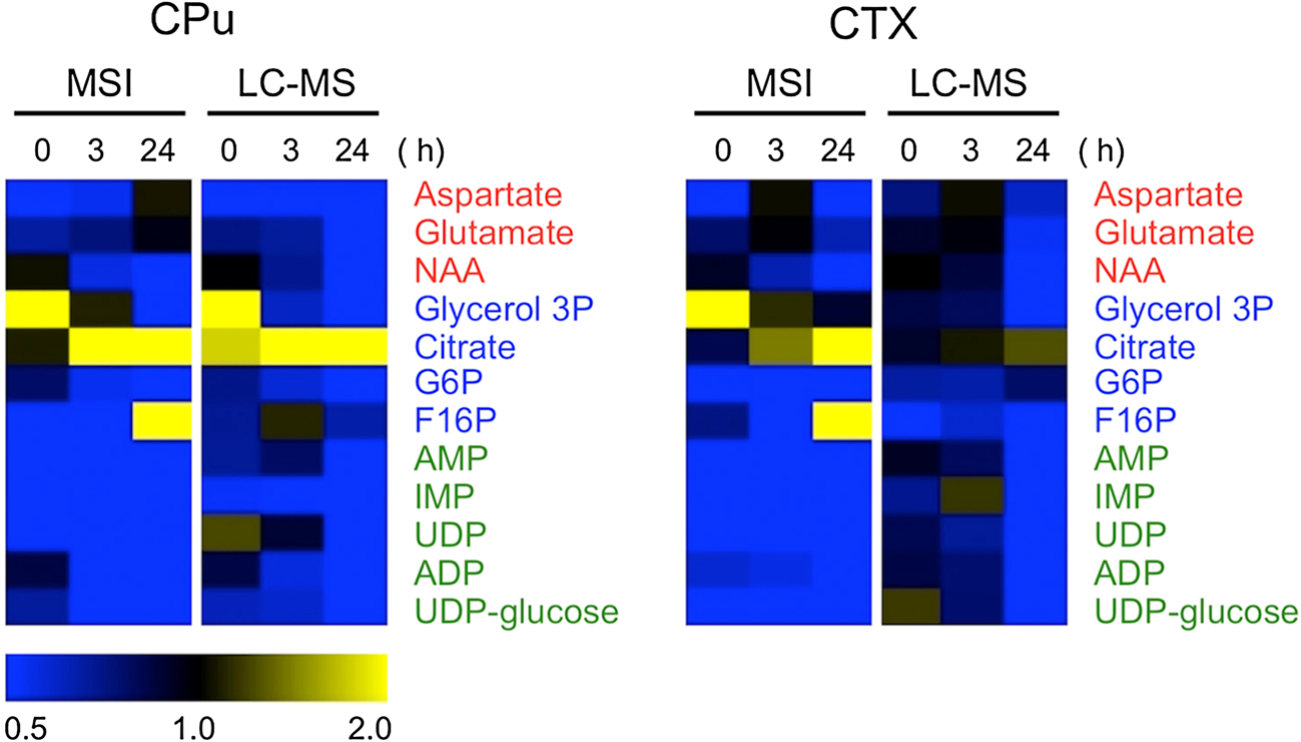
A comparison of the average intensity between the whole tissue regions and partial tissue regions. The average intensity of the whole tissue region is represented as the average intensity ratio (ischemic hemisphere/contralateral hemisphere) for each whole tissue region (LC–MS data, see Fig. 1a). The regional average intensity of a partial tissue region is represented as the average intensity of each region on 10 μm slices (MSI data, see Fig. 1b). In the heat map, 12 common metabolites detected by LC–MS and MSI were visualized. The normalized mean values were displayed using the MultiExperiment Viewer (http://www.tm4.org). The colored letters for the heat map are as follows: amino acids (*red*), central metabolism intermediates (*blue*), nucleic acids (*green*), other metabolites (*orange*)

The data for 12 metabolites commonly detected by both LC–MS and MSI were examined by a heat map analysis (Fig. 4). Although some partial MSI data were already published (Miura et al. 2010b), a further data analysis was performed in this study. Each data was expressed as the ratio of the Isc. hemisphere to the Con. hemisphere. Similar behaviors of several metabolite levels were observed between the MSI and LC–MS data, although the intensity ratios were different. In particular, the time-dependent changes in *N*-acetyl aspartate (NAA) were similar to those of glycerol-3-phosphate (glycerol-3P) and citrate in the CPu and CTX regions in both analytical platforms.

On the other hand, the correlations between the data acquired by two different platforms were low for fructose 1,6-bisphosphate (F16P) (CPu and CTX), glutamate and aspartate (CPu), and UDP-glucose (CTX). The coronally sliced tissue samples of MSI contained the central portions of the ischemic lesions, and the obtained data directly reflect the metabolic change during ischemia–reperfusion in the tissue micro-region level (i.e., highly resolved spatial information of the target region). In contrast, the data from LC–MS were averaged information of the whole region that contained the non-ischemic area. Therefore, the different metabolic events between the analytical platforms may be due to the different metabolic changes between the affected area and its peripheral area. The temporal expression patterns of citrate and glycerol-3P after reperfusion in the CPu and CTX were highly correlated between the MSI and LC–MS data. In the CPu, such metabolite concentrations were similar between the platforms, although these concentrations were clearly different in the CTX. The metabolism in the whole region of the CPu is relatively homogeneously regulated by the MCA, whereas many micro-regions of the CTX are not. We speculate that the differences in the regulation of blood flow between CTX and CPu regions may have contributed to the differences between the LC–MS and MSI data. The LC–MS data at least relatively reflected the time-dependent metabolic changes shown by MSI. Therefore, the information about the metabolites detected by LC–MS, but not by MSI, may lead to an understanding of the spatial characteristics of the metabolic state in response to pathological progression.

### Spatiotemporal metabolomic dynamics and metabolic pathway analysis by combing MSI and LC–MS

**Fig. 5:**
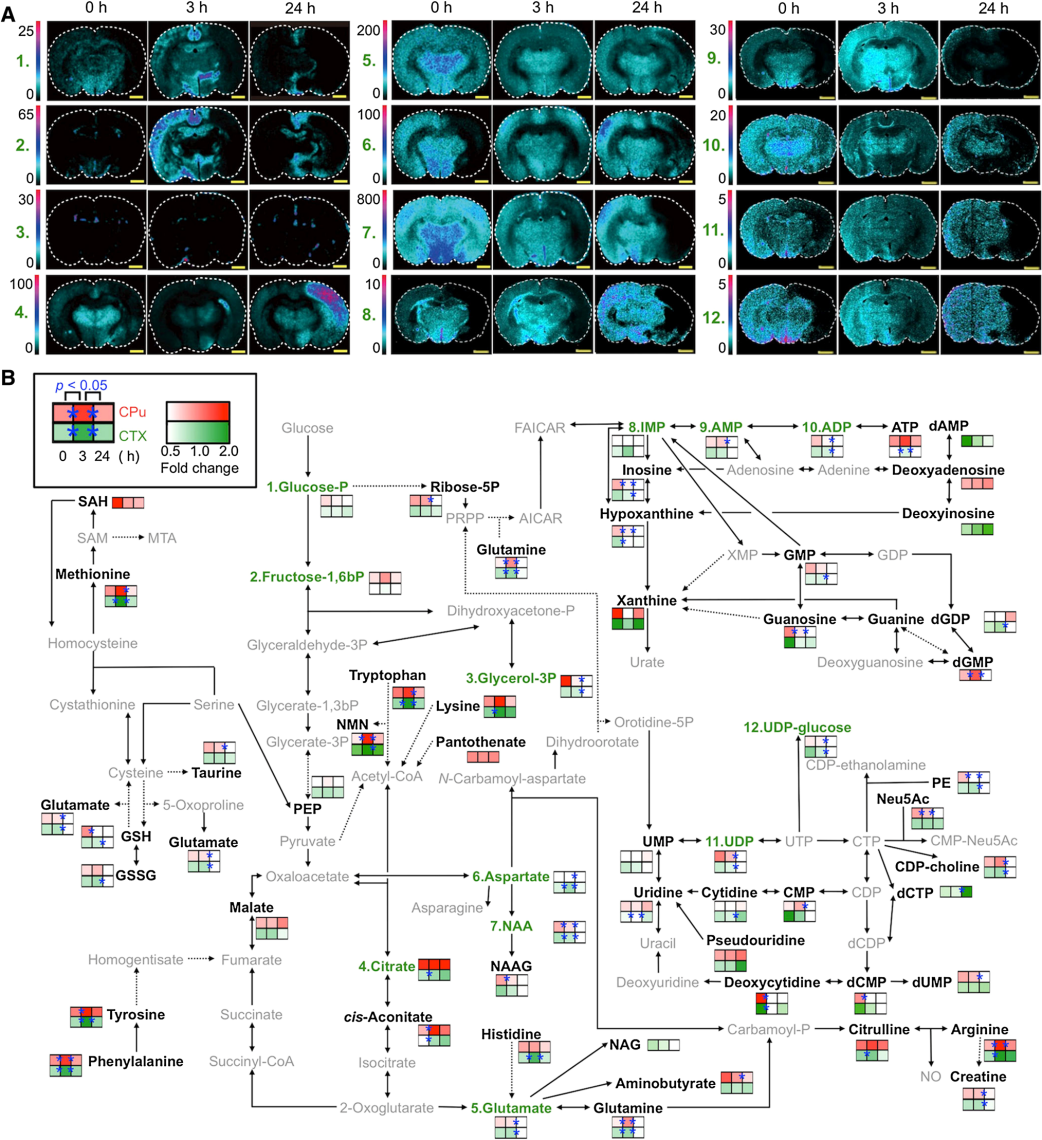
The integrated MSI and LC–MS techniques allow the visualization of drastic changes in the spatiotemporal metabolite distribution. **a** In situ MSI visualized dramatic changes in the spatiotemporal metabolite distribution in the MCAO rat brain. Metabolites related to nucleotide and amino acid metabolism, as well as the central pathway, were simultaneously visualized in a single MSI experiment. *Scale bar* 1.0 mm. Data (No. 1–7) were reprinted with permission from the author (Miura et al. 2010b). **b** Comparative visualization of the central metabolic pathway and its peripheral metabolic pathways in the CPu (*upper box*, *red*) and CTX (*lower box*, *green*) in the MCAO rat brain, as determined by LC–MS. Significant differences (Student’s *t* test, *P < 0.05) are indicated by *asterisks* on the *colored boxes*. *Black* and *grey letters* indicate LC–MS-detected metabolites and unmeasured metabolites, respectively. *Green letters* indicate metabolites detected by both LC–MS and MSI. *Solid arrows* represent a single step connecting two metabolites, and *dotted arrows* represent multiple steps

As described above, we found several properties of both MS platforms in the metabolomic analysis. To expand this knowledge to a pathological evaluation, we applied this technique to visualize more comprehensive spatiotemporal metabolic dynamics in the pathogenesis of ischemia–reperfusion injury (Fig. 5). The MSI data, indicating the time-dependent changes in the metabolite distribution at 0, 3 and 24 h after reperfusion, are shown in Fig. 5a. A quantitative comparison (the ratio of the Isc. hemisphere to the Con. hemisphere) of time-dependent metabolite variations in molecules related to glycolysis, the TCA cycle, nucleotide and amino acid metabolism in the CPu and CTX was performed by using LC–MS (Fig. 5b). By integrating the highly quantitative data obtained from LC–MS and the spatially resolved data obtained from MSI, we could investigate the spatiotemporal metabolic variance.

With regard to the pyrimidine metabolism, in the MSI analysis, a significant decrease in uridine diphosphate (UDP) and UDP-glucose was observed over the whole region of the Isc. hemisphere 24 h after reperfusion (Fig. 5a, No. 11 and 12). In the LC–MS analysis, a reduction of UDP derivatives (UDP and UDP-glucose), as well as peripheral metabolites (cytidine diphosphate (CDP)-choline, phosphoethanolamine (PE), and *N*-acetylneuraminate (Neu5Ac)), was observed at 24 h. Previously, it was reported that the production of ROS was increased during ischemia, and its level was further elevated with the onset of reperfusion, and finally, the generated ROS were able to cause injury to the plasma membranes, thus leading to cell death (Bromont et al. 1989). The reduced levels of membrane intermediates, such as PE, CDP-choline, UDP and UDP-glucose, may have been due to their consumption for plasma membrane synthesis to restore the membrane integrity following ROS-mediated damage.

The levels of many amino acids, the catabolites of proteins, were increased, except for glutamate, which is related to various metabolic reactions, after 3 h of reperfusion in the Isc. hemisphere (Fig. 5b). The inhibition of protein synthesis and the progression of protein degradation after reperfusion have been reported previously (Bylund-Fellenius et al. 1984; Neumar 2000). Although MSI data alone cannot explain the characteristic behavior of amino acids, the above-mentioned protein alterations after reperfusion probably contribute to the accumulation of the building blocks of proteins 3 h after reperfusion. MSI also showed a significant increase in the aspartate level in the whole regions of the Isc. hemisphere after 3 h of reperfusion (Fig. 5a, No.6). In this study, MSI was not able to trace most of the amino acids measured by LC–MS. However, our findings that the LC–MS data exhibited a relatively high correlation with the MSI data (Fig. 4) suggest that amino acids showing a similar behavior to aspartate in the LC–MS results may show an aspartate-like distribution in the Isc. hemisphere, although further studies are needed to confirm whether this is the case.

On the other hand, the levels of almost all amino acids, including glutamate, were reduced at 24 h (Fig. 5b). The energy production was reported to be depressed after reperfusion due to the inactivation of TCA cycle-related enzymes, aconitase and 2-oxoglutarate dehydrogenase, resulting from the hypergeneration of ROS (Tretter and Adam 2000). In addition, we previously found that citrate synthase activity was not affected by ischemia–reperfusion (Miura et al. 2010b). The LC–MS analysis in the present study also showed that TCA cycle intermediates, citrate (CPu and CTX) and malate (CPu), were accumulated in the Isc. hemisphere after reperfusion. These observations raise a possibility that the reduction of amino acids may be partially due to their utilization as TCA cycle intermediates for reactivation of the TCA cycle. The MSI data also revealed that the increase in citrate, as well as the decrease in aspartate and glutamate after 24 h of reperfusion were characteristically heterogeneously distributed in the Isc. hemisphere (Fig. 5a, No. 4–6).

As previously mentioned in the description of the LC–MS-based metabolic profiling (Fig. 2), the blood supply of the brain is provided via not only the MCA, but also several other arteries (such as the anterior and posterior cerebral arteries). The artery-regulated region is not necessarily consistent with each functional compartment. In fact, the blood supply of the CTX is regulated by both the MCA and other arteries. Intriguingly, the region with the most characteristic metabolic variation (citrate, aspartate and glutamate) shown by MSI was relatively consistent with the specific region demonstrating remarkable lowering of the blood flow by occlusion of the MCA (Kitagawa et al. 1998).

## Concluding remarks

In the present study, we were able to visualize diverse spatiotemporal metabolic dynamics within the central metabolic pathway and its periphery in the MCAO rat brain during infarct formation using an integrated technique combining MSI with LC–MS. Using representative tissue samples of different functional compartments, LC–MS was used to trace the temporal changes of many metabolites which could not be visualized by MSI. Significant metabolic changes in the blood supplying area from the MCA were observed by a series of region-specific behavioral analyses. To date, conventional pathological analysis of MCAO models has not shown the spatiotemporal metabolic behaviors after reperfusion. In contrast, we herein visualized a MCA-regulated metabolic change in several metabolic pathways, including the pyrimidine-, amino acid- and TCA cycle-related metabolism, during pathological progression. Thus, our new approach combining MSI (spatial information but low coverage) and its complementary technique LC–MS (high coverage but loss of spatial information) will provide insight into the understanding of the complex pathological mechanism(s) of ischemia–reperfusion injury. This could be broadly applicable to emerging issues in the precise pathological evaluation of target tissues in both preclinical and clinical settings, and may become a compulsory technique for in situ pharmacometabolomics and biomarker discovery.

## Electronic supplementary material

Below is the link to the electronic supplementary material.

Supplementary material 1 (DOC 3380 kb)

## Abbreviations

9-AA: 9-Aminoacridine
ADP: Adenosine diphosphate
AICAR: 5-Aminoimidazole-4-carboxamide ribonucleotide
AMP: Adenosine monophosphate
ATP: Adenosine triphosphate
CDP: Cytidine diphosphate
CMP: Cytidine monophosphate
Con.: Contralateral
CPu: Corpus striatum
CTP: Cytidine triphosphate
CTX: Cortex
dAMP: Deoxy-AMP
dCMP: Deoxy-CMP
dCTP: Deoxy-CTP
dGDP: Deoxy-GDP
dGMP: Deoxy-GMP
dUMP: Deoxy-UMP
F16P: Fructose 1,6-bisphosphate
FAICAR: 5-Formamidoimidazole-4-carboxamide ribotide
G6P: Glucose monophosphate
GDP: Guanosine diphosphate
glycerol-3P: Glycerol 3-phosphate
GMP: Guanosine monophosphate
GSH: Glutathione-SH
GSSG: Glutathione-*S*-*S*-glutathione
HI: Hippocampus
IMP: Inosine monophosphate
Isc.: Ischemic
LC: Liquid chromatography
MALDI-TOF: Matrix-assisted laser desorption and ionization time of flight
MCA: Middle cerebral artery
MCAO: MCA occlusion
MS: Mass spectrometry
MSI: MS imaging
MTA: 5′-Methylthioadenosine
NAA: *N*-acetyl aspartate
NAAG: *N*-acetylaspartylglutamate
NAD+: Nicotinamide adenine dinucleotide
NADPH: Nicotinamide adenine dinucleotide phosphate
NAG: *N*-acetylglutamate
Neu5Ac: *N*-acetylneuraminate
NO: Nitric oxide
PCA: Principal component analysis
PE: Phosphoethanolamine
PEP: Phosphoenolpyruvate
PRPP: Phosphoribosyl pyrophosphate
ribose-5P: Ribose 5-phosphate
ROS: Reactive oxygen species
SAH: *S*-adenosyl-l-homocysteine
SAM: *S*-adenosylmethionine
UDP: Uridine diphosphate
UMP: Uridine monophosphate
UTP: Uridine triphosphate
XMP: Xanthosine monophosphate
