# Supplementary material for: Integrated MALDI-MS imaging and LC–MS techniques for visualizing spatiotemporal metabolomic dynamics in a rat stroke model
Source: Metabolomics. 2013 Oct 13;10(3):473–83. doi: 10.1007/s11306-013-0588-8 (PMC3984668; doi:10.1007/s11306-013-0588-8)
Supplement: Supplementary file 1 — Supplementary material 1 (DOC 3380 kb) [file 11306_2013_588_MOESM1_ESM.doc]

Supporting Information

Integrated MALDI-MS Imaging and LC-MS Techniques for Visualizing Spatiotemporal Metabolomic Dynamics in a Rat Stroke Model

Miho Irie^1^, Yoshinori Fujimura^2^, Mayumi Yamato ^2^,Daisuke Miura ^2, *^, Hiroyuki Wariishi ^2, 3, 4,*^

^1^Graduate School of Bioresource and Bioenvironmental Sciences, Kyushu University, ^2^Innovation Center for Medical Redox Navigation, Kyushu University, ^3^Bio-architecture Center, Kyushu University, and ^4^Faculty of Arts and Science, Kyushu University

Contents

(1) Table S1 Mass-to-charge ratio, molecular species and fragments observed on tissue sections.

(2) Fig. S1 Experimental procedures for the LC-MS and MSI analyses.

(3) Fig. S2 Schematic illustrations of the functional regions in the cortex.

(4) Fig. S3 The LC-MS analysis of region-specific metabolomic changes during reperfusion.

(5) Fig. S4 The LC-MS analysis of region-specific metabolomic changes during reperfusion.

(6) Fig. S5 The LC-MS analysis of region-specific metabolomic changes during reperfusion.

(7) Fig. S6 The LC-MS analysis of region-specific metabolomic changes during reperfusion.

**Supplemental Figure Legends**

**Table S1** **Mass-to-charge ratio, molecular species and fragments observed on tissue sections.**

**Fig. S1 Experimental procedures for the LC-MS and MSI analyses.**

(A) A schematic representation of the LC-MS analysis workflow. (B) An overview of the MSI analysis procedure.

**Fig. S2 Schematic illustrations of the functional regions in the cortex.**

A schematic illustration represents the structure of coronally sectioned rat brain. Different parts of the cerebral cortex are involved in different cognitive and behavioral functions.

**Fig. S3 The LC-MS analysis of region-specific metabolomic changes during reperfusion.**

The data indicate the average intensity (ischemic hemisphere/contralateral hemisphere) of amino acid metabolism related metabolites at each whole tissue region (CPu or CTX). The values are the means of five replicates + SD. Asterisks indicate significant differences between 0 and 3 h, or 3 and 24 h, as determined by Student’s *t*-test (**P* < 0.05; ***P* < 0.01).

**Fig. S4 The LC-MS analysis of region-specific metabolomic changes during reperfusion.**

The data indicate the average intensity (ischemic hemisphere/contralateral hemisphere) of central metabolism related metabolites at each whole tissue region (CPu or CTX). The values are the means of five replicates + SD. Asterisks indicate significant differences between 0 and 3 h, or 3 and 24 h, as determined by Student’s *t*-test (**P* < 0.05; ***P* < 0.01).

**Fig. S5 The LC-MS analysis of region-specific metabolomic changes during reperfusion.**

The data indicate the average intensity (ischemic hemisphere/contralateral hemisphere) of nucleotide metabolism related metabolites at each whole tissue region (CPu or CTX). The values are the means of five replicates + SD. Asterisks indicate significant differences between 0 and 3 h, or 3 and 24 h, as determined by Student’s *t*-test (**P* < 0.05; ***P* < 0.01).

**Fig. S6 The LC-MS analysis of region-specific metabolomic changes during reperfusion.**

The data indicate the average intensity (ischemic hemisphere/contralateral hemisphere) of metabolites except for amino acid, central, and nucleic acid metabolism related metabolites at each whole tissue region (CPu or CTX). The values are the means of five replicates + SD. Asterisks indicate significant differences between 0 and 3 h, or 3 and 24 h, as determined by Student’s *t*-test (**P* < 0.05; ***P* < 0.01).

**Table S1**

| *m/z* | *Molecular species* | *Fragments observed* | *Formula* |
| --- | --- | --- | --- |
| 426 | ADP | 328, 291, 159, 134, 79 | C_10_H_15_N_5_O_10_P_2_ |
| 346 | AMP | 211, 151, 97 | C_10_H_14_N_5_O_7_P |
| 339 | F1,6P | 242, 160, 97, 79 | C_6_H_14_O_12_P_2_ |
| 347 | IMP | 211, 151, 135, 97, 92, 79 | C_10_H_13_N_4_O_8_P |
| 565 | UDP-glucose | 403, 385, 323, 305, 241, 159, 97, 79 | C_15_H_24_N_2_O_17_P_2_ |
| 403 | UDP | 323, 305, 291, 159, 111, 97 | C_9_H_14_N_2_O_12_P_2_ |
| 132 | aspartate | 115, 88 | C_4_H_7_NO_4_ |
| 191 | citrate | 134, 131, 111 | C_6_H_8_O_7_ |
| 259 | G6P | 199, 169, 97, 79 | C_6_H_13_O_9_P |
| 146 | glutamate | 128, 102 | C_5_H_9_NO_4_ |
| 171 | Glycerol-P | 97, 79 | C_3_H_9_O_6_P |
| 174 | NAA | 130, 114, 58 | C_6_H_9_NO_5_ |

**
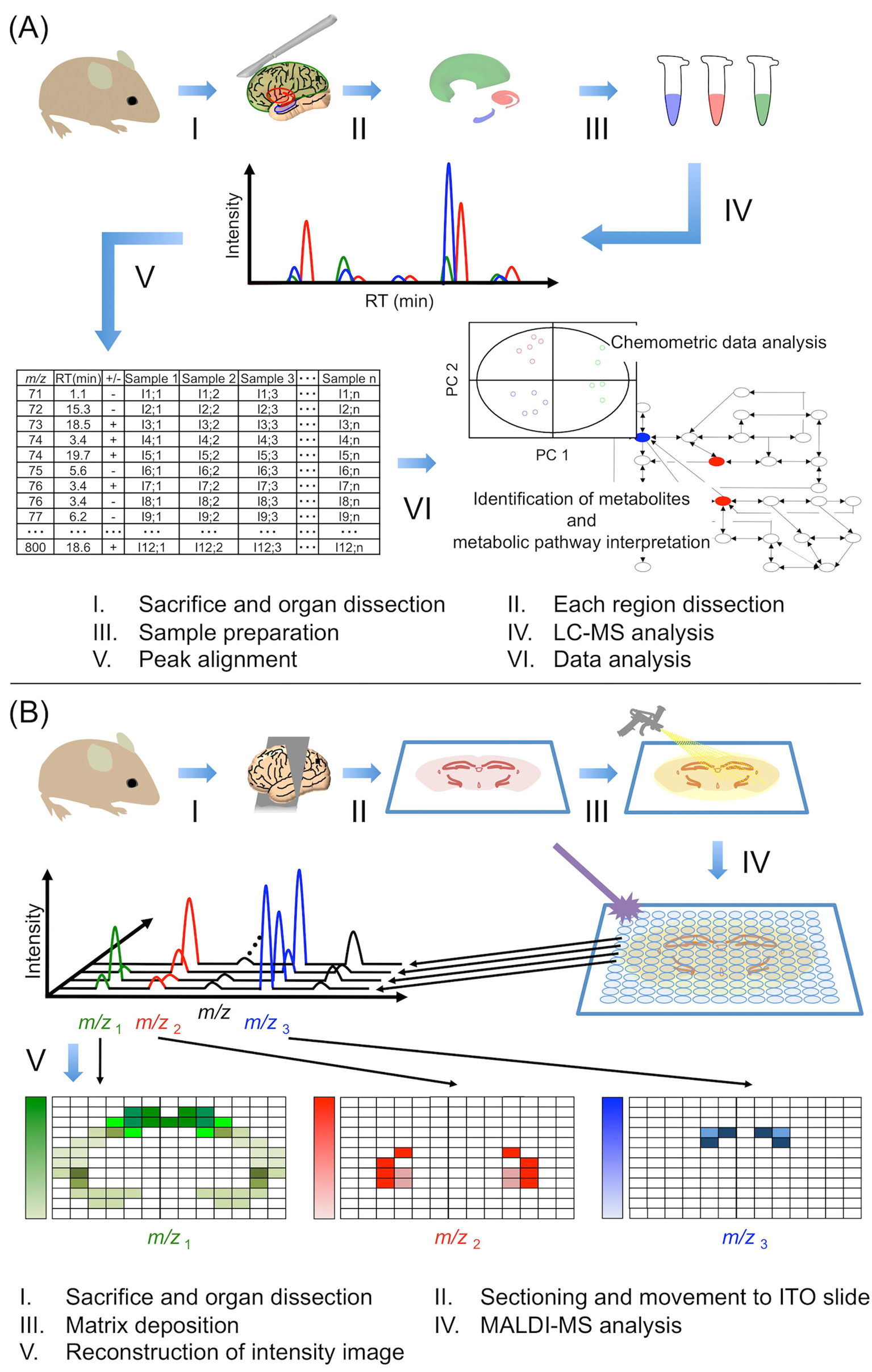
**

**Figure S1**


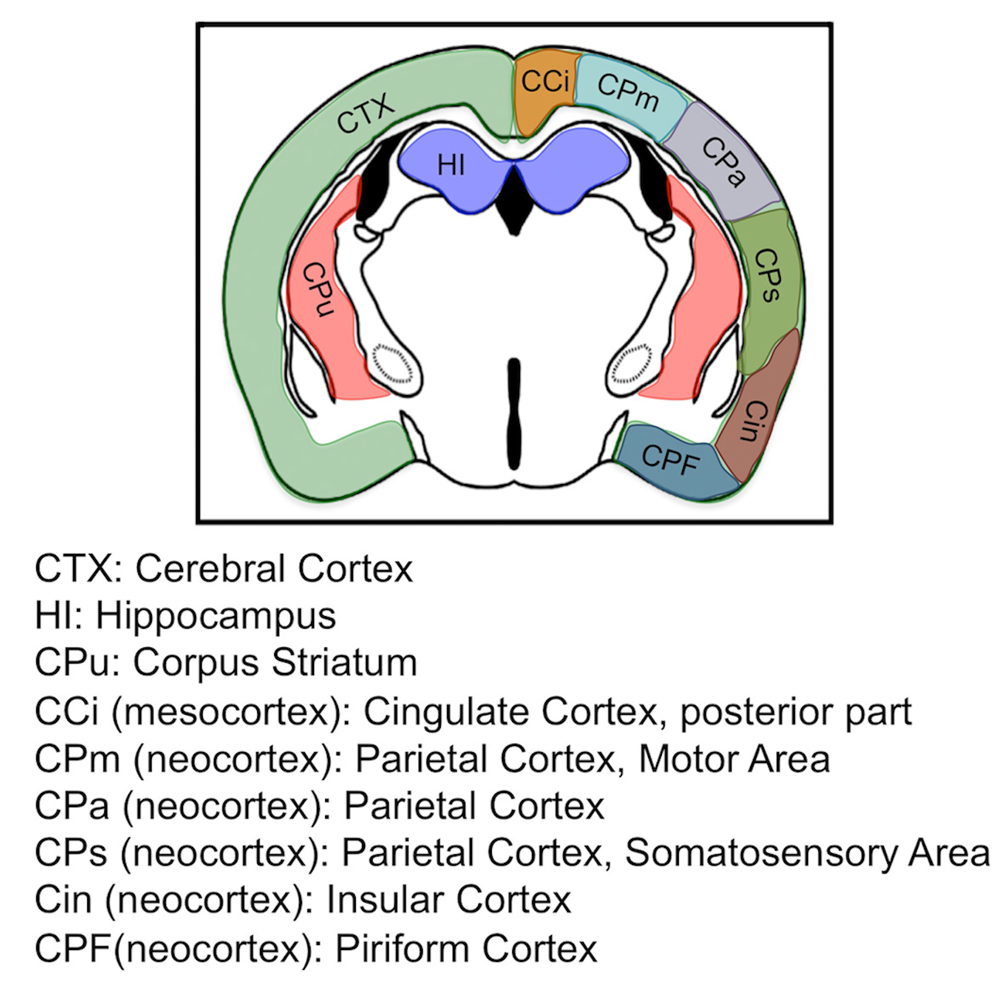


**Figure S2**

**
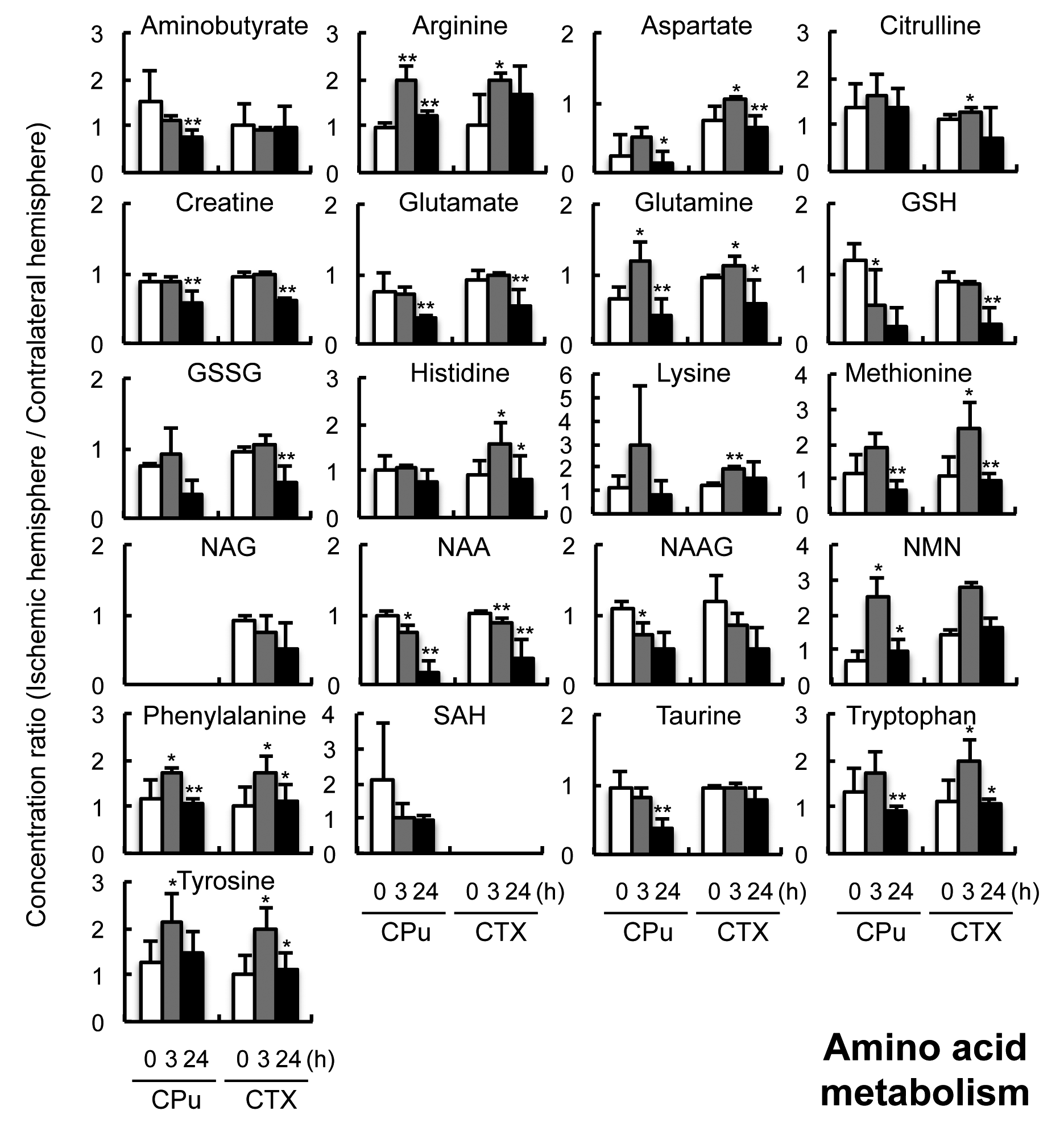
**

**Figure S3**


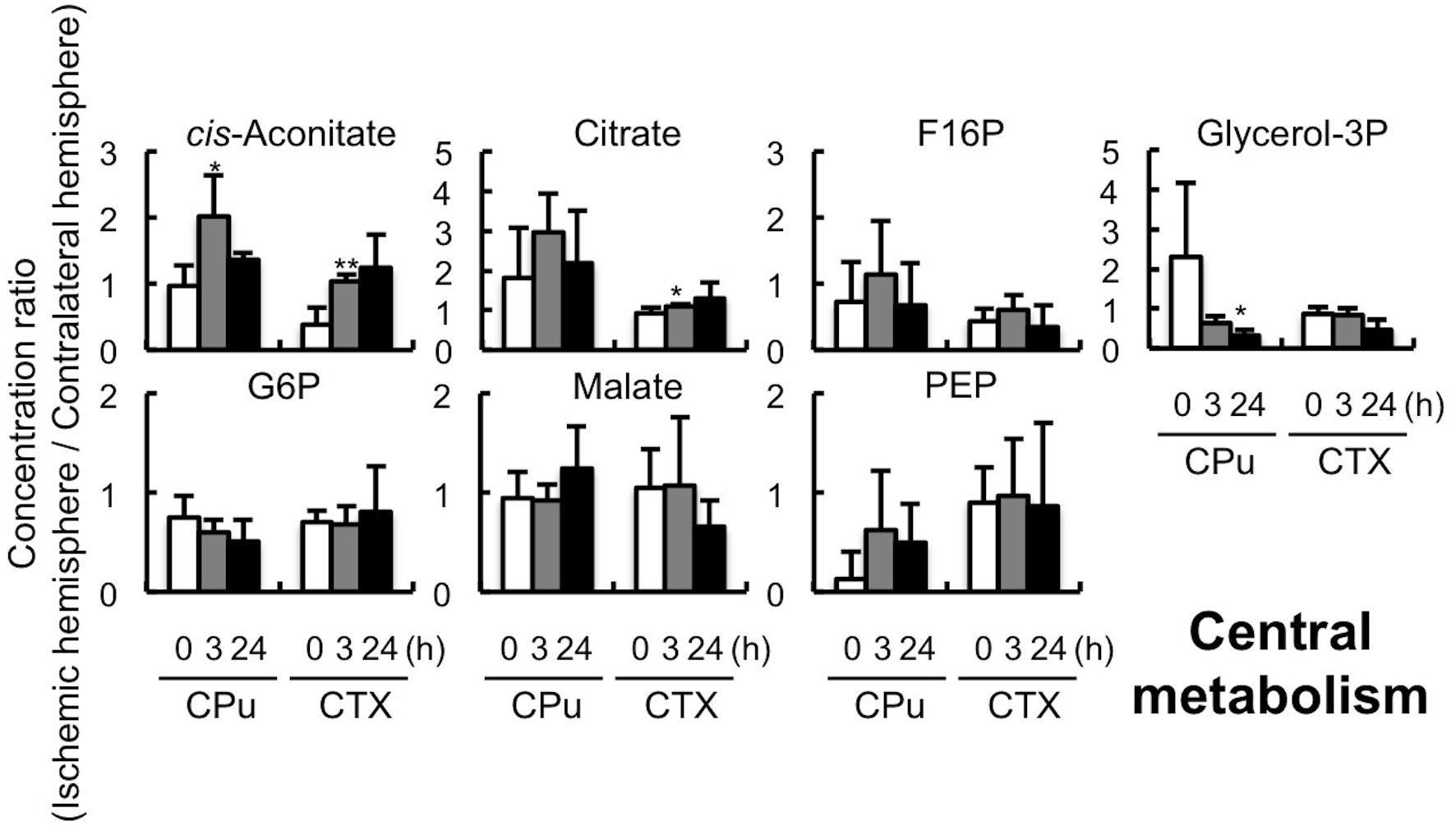


**Figure S4**

**Figure S5**

**
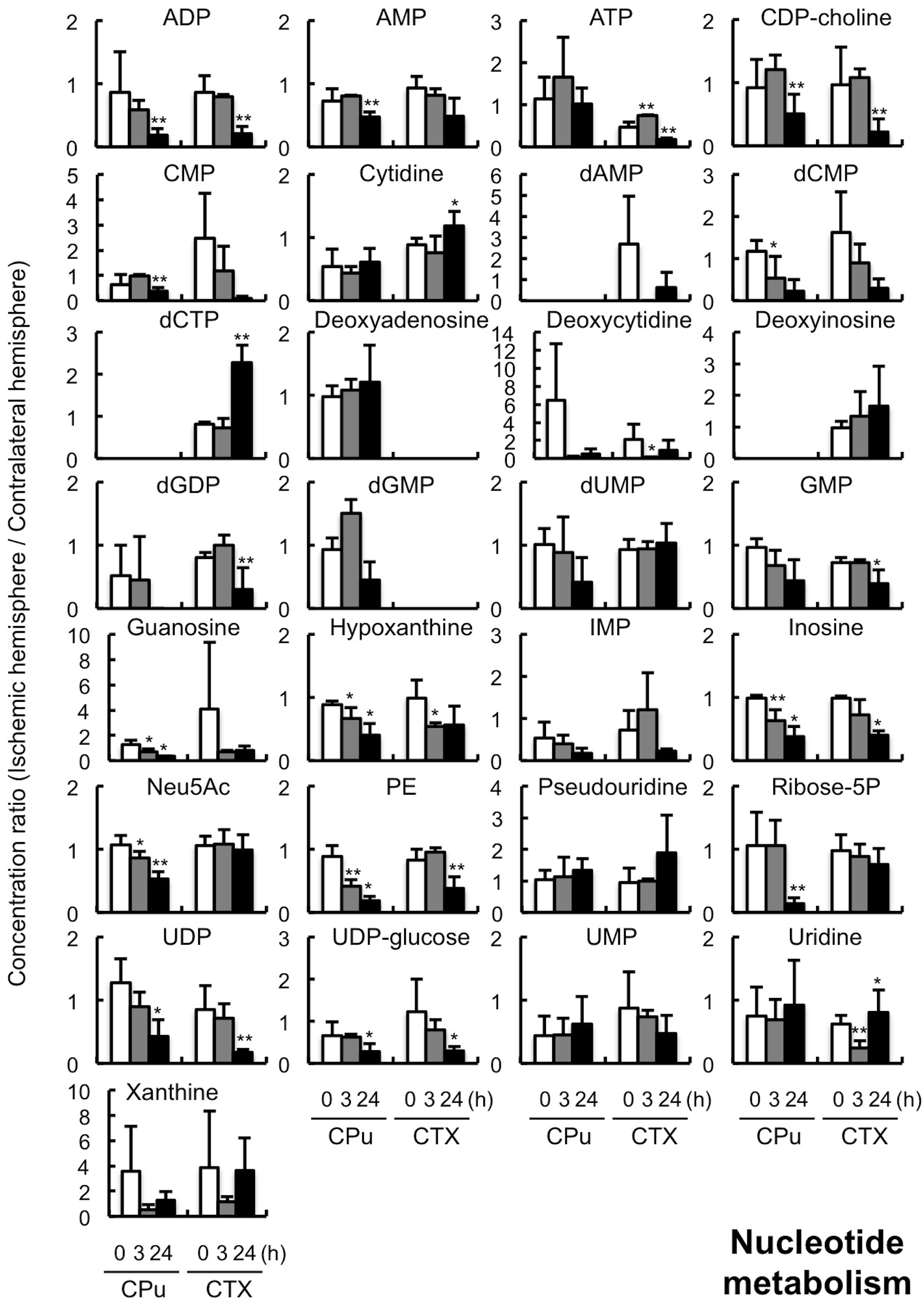
**

**
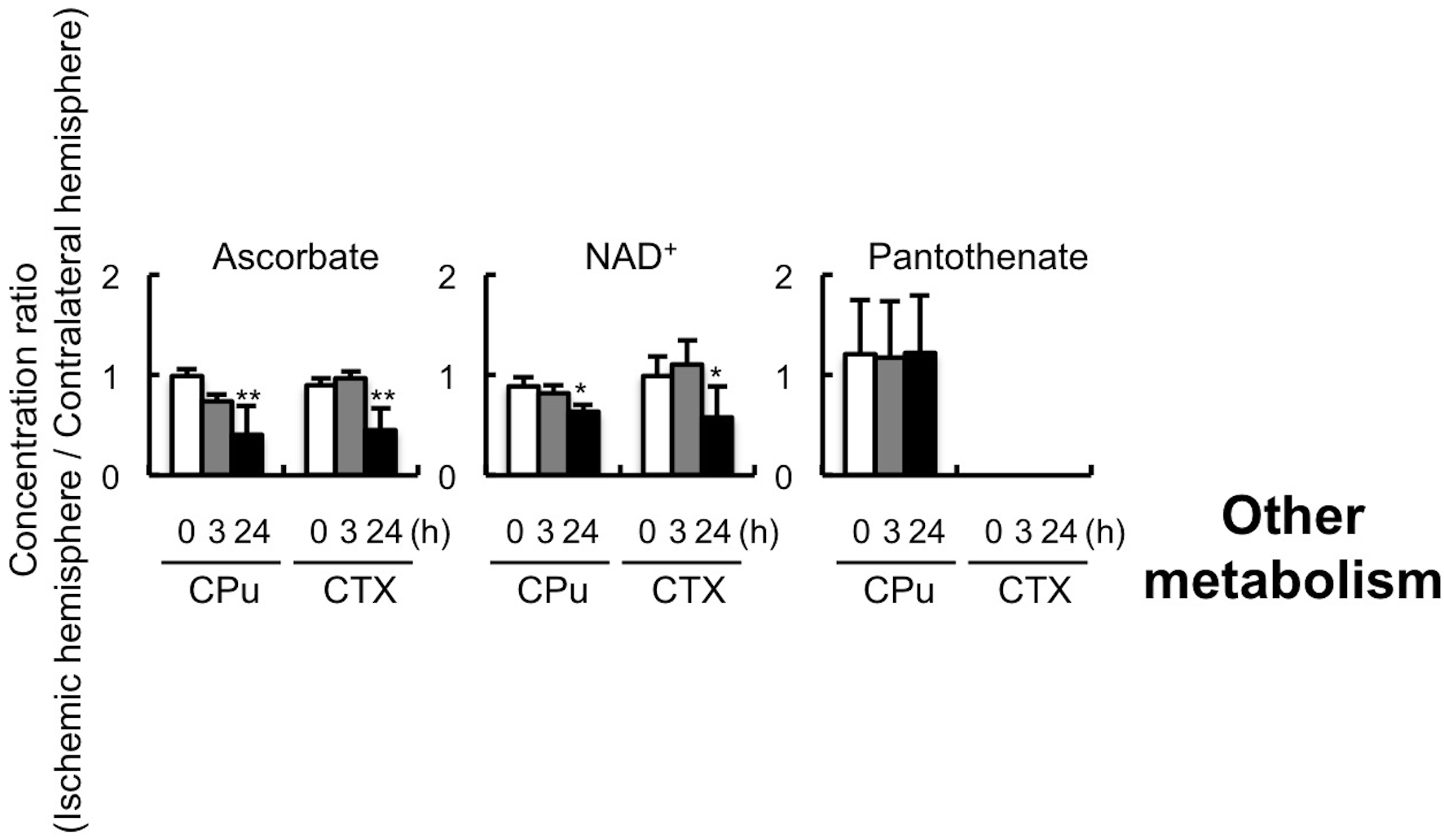
**

**Figure S6**
